# Public Surveillance of Social Media for Suicide Using Advanced Deep Learning Models in Japan: Time Series Study From 2012 to 2022

**DOI:** 10.2196/47225

**Published:** 2023-06-02

**Authors:** Siqin Wang, Huan Ning, Xiao Huang, Yunyu Xiao, Mengxi Zhang, Ellie Fan Yang, Yukio Sadahiro, Yan Liu, Zhenlong Li, Tao Hu, Xiaokang Fu, Zi Li, Ye Zeng

**Affiliations:** 1 Graduate School of Interdisciplinary Information Studies University of Tokyo Tokyo Japan; 2 School of Earth and Environmental Sciences, The University of Queensland Brisbane Australia; 3 School of Science, RMIT University Melbourne Australia; 4 Department of Geography University of South Carolina Columbia, SC United States; 5 Department of Geosciences University of Arkansas Fayetteville, AR United States; 6 Department of Population Health Sciences Weill Cornell Medicine New York, NY United States; 7 Carilion School of Medicine Virginia Tech Blacksburg, VA United States; 8 School of Communication and Mass Media Northwest Missouri State University Maryville, MO United States; 9 School of Earth and Environmental Sciences University of Queensland Brisbane Australia; 10 Department of Geography Oklahoma State University Stillwater, OK United States; 11 Centre for Geographic Analysis Harvard University Cambridge, MA United States; 12 Graduate School of Medicine Juntendo University Tokyo Japan; 13 Department of Medical Business Nihon Pharmaceutical University Tokyo Japan

**Keywords:** suicide, suicidal ideation, suicide-risk identification, natural language processing, social media, Japan

## Abstract

**Background:**

Social media platforms have been increasingly used to express suicidal thoughts, feelings, and acts, raising public concerns over time. A large body of literature has explored the suicide risks identified by people’s expressions on social media. However, there is not enough evidence to conclude that social media provides public surveillance for suicide without aligning suicide risks detected on social media with actual suicidal behaviors. Corroborating this alignment is a crucial foundation for suicide prevention and intervention through social media and for estimating and predicting suicide in countries with no reliable suicide statistics.

**Objective:**

This study aimed to corroborate whether the suicide risks identified on social media align with actual suicidal behaviors. This aim was achieved by tracking suicide risks detected by 62 million tweets posted in Japan over a 10-year period and assessing the locational and temporal alignment of such suicide risks with actual suicide behaviors recorded in national suicide statistics.

**Methods:**

This study used a human-in-the-loop approach to identify suicide-risk tweets posted in Japan from January 2013 to December 2022. This approach involved keyword-filtered data mining, data scanning by human efforts, and data refinement via an advanced natural language processing model termed Bidirectional Encoder Representations from Transformers. The tweet-identified suicide risks were then compared with actual suicide records in both temporal and spatial dimensions to validate if they were statistically correlated.

**Results:**

Twitter-identified suicide risks and actual suicide records were temporally correlated by month in the 10 years from 2013 to 2022 (correlation coefficient=0.533; *P*<.001); this correlation coefficient is higher at 0.652 when we advanced the Twitter-identified suicide risks 1 month earlier to compare with the actual suicide records. These 2 indicators were also spatially correlated by city with a correlation coefficient of 0.699 (*P*<.001) for the 10-year period. Among the 267 cities with the top quintile of suicide risks identified from both tweets and actual suicide records, 73.5% (n=196) of cities overlapped. In addition, Twitter-identified suicide risks were at a relatively lower level after midnight compared to a higher level in the afternoon, as well as a higher level on Sundays and Saturdays compared to weekdays.

**Conclusions:**

Social media platforms provide an anonymous space where people express their suicidal thoughts, ideation, and acts. Such expressions can serve as an alternative source to estimating and predicting suicide in countries without reliable suicide statistics. It can also provide real-time tracking of suicide risks, serving as an early warning for suicide. The identification of areas where suicide risks are highly concentrated is crucial for location-based mental health planning, enabling suicide prevention and intervention through social media in a spatially and temporally explicit manner.

## Introduction

Suicide is the 17th leading cause of death worldwide [1]. It affects people of all ages globally but is more severe in some regions and populations. In Japan, suicide is the top-leading cause of death in people aged 15-34 years for both sexes [2]. Despite the devastating and inevitable impact, many suicide behaviors are preventable. Based on a cross-national study, about 60% of suicidal ideation transitions to suicide plans, and suicide attempts occurred in the first year after the onset of suicidal ideation [3]. Thus, timely interventions for those with suicidal thoughts are crucial to preventing suicide. However, many of those with suicidal ideation do not express themselves nor do they look for help from professional health care providers due to stigma, the fear of loss of autonomy, and overreaction by others [4]. Those barriers make suicide difficult to predict and prevent. With the dramatically growing number of social media users, suicide risk information has been increasingly expressed on various social media platforms. Recent studies discovered that web-based social media data, particularly tweets, include predictive suicide ideation data that may be used for suicide prevention [5-7]. However, less evidence has been provided to corroborate whether suicide risks identified on social media aligns with actual suicidal behaviors in terms of timing and location. If there is alignment, social media platforms could be better designated for suicide prevention and serve as an alternative source to estimating and predicting suicide, especially in countries without reliable suicide statistics.

There is a growing body of literature using social media data and advanced techniques (eg, machine learning and deep learning models) to identify suicide risks and improve suicide prevention [8,9]. However, there are at least three areas of knowledge deficits in the current scholarship. First, the existing studies are predominantly based on English-only social media contents [5,10], possibly due to the technical barriers that the majority of natural language processing models do not have the capacity to cope with multilingual contents. Second, the mainstream of current studies focuses on identifying suicide ideation, thoughts, or acts [11] on social media but few studies link them to the actual suicide behaviors—such a linkage would provide sufficient evidence to support the efficiency of social media platforms for tracking and preventing suicide. Third, few studies give attention to long-term tracking of suicide risks in terms of timing and location to empower mental health planning that is temporally and spatially explicit [12]. In the context of Japan, a super-aging society with a high suicide rate among high-income countries, the English literature on suicide is somehow limited compared to its counterparts. Although a few studies use social media data to monitor suicide in Japan [13-15], they do not have wide spatial and temporal coverage nor do they contrast the suicide risks identified in social media with reality—the gap to be fulfilled in this study.

This study aimed to corroborate whether suicide risks identified on social media align with actual suicidal behaviors by tracking suicide risks detected from 62 million tweets posted in Japan from January 2013 to December 2022 and compare such suicide risks with actual suicide statistics in terms of their timing and location. We took Twitter as the data source to retrieve text-based contents given that Twitter is one of the most popular social media platforms with the largest number of users in Japan, where people express their ideas, opinions, and perceptions [16]. Twitter data have been consistently used in suicide-related studies in the Japanese context [14,15]. To identify the suicide risk tweets, we used a human-in-the-loop approach, involving keyword-filtered data mining, data scanning by human efforts, and data refinement via an advanced natural language processing model termed Bidirectional Encoder Representations from Transformers (BERT). Then, the tweet-identified suicide risks were compared with actual suicide records in both temporal and spatial dimensions to validate if these 2 measures are statistically correlated. Our findings provide evidence over 10 years in Japan to support that social media provides public surveillance for suicide. It serves as an alternative source to estimating and predicting suicide in countries without reliable suicide statistics and enables real-time tracking of suicide risks. Delineating areas where suicide risks are highly concentrated is crucial for location-based mental health planning, enabling suicide prevention and intervention through social media that are spatially and temporally explicit.

## Methods

### Data Collection

We retrieved tweets via Twitter academic application programming interface (API) based on the following criteria: (1) the tweets were posted in Japan in the Japanese language; (2) the time of posting was between January 1, 2013, and December 31, 2022; (3) the tweets contained an array of keywords that include suicide risk information (Table S1 in Multimedia Appendix 1); and (4) retweets were excluded. Referring to relevant studies in both English and Japanese [5,13,17,18], we compared the keywords commonly used in both literature and found that some keywords appearing in the English literature (eg, “gun shoot” and “shoot myself”) did not apply to the Japanese context. After calibration and validation, we eventually identified 55 keywords that commonly appear in suicide-related tweets (Table S1 in Multimedia Appendix 1). Such keywords included (1) terms with “suicid*” (eg, “suicidal” or “suicide site”); (2) jargon and slang of suicide (eg, “leave this world”); (3) suicidal ideation (eg, “want to end up my life”); (4) suicidal behaviors (eg, “burn myself”); or (5) suicide-relevant psychiatric symptoms (eg, “depression” or “euthanasia”). We extracted a total of 62,083,647 tweets that were posted in Japan from 2013 to 2022 containing suicide-related keywords (Table S2 in Multimedia Appendix 1). This tweets data set included user ID, conversation ID, time when a tweet was posted, languages used, latitude and longitude of the place where a tweet was posted (although only a small number of tweets contained such locational information), place names (ie, a city, country, or both where a tweet was posted), and texts (ie, the content of the tweets). We further refined the tweets that contain geographic information (ie, the latitude and longitude of a place or the name of a place) to forge a geotweet data set (a total of 23,815 tweets, accounting for 0.038% of the 62,083,647 total tweets; see Table S3 in Multimedia Appendix 1).

Furthermore, we collected the total number of all tweets posted in Japanese in each month from 2013 to 2022 based on the Japanese keywords “ノ” and “の,” as they are the most frequently used words in Japanese [19]. In doing so, we assumed that tweets containing these 2 keywords approximately represent the total tweets posted in a certain month since the API needs at least one predefined keyword to retrieve tweets. Given that the number of retweets changed substantially over the years with increased active Twitter users from 2013 to 2022, we calculated the total number of tweets, including and excluding retweets separately (Table S3 in Multimedia Appendix 1).

In addition, we collected the actual suicide records provided in the suicide statistics published by the Ministry of Health, Labour, and Welfare [20]. This actual suicide data set contains city-by-month records of suicide deaths among all citizens in Japan from January 2013 to December 2022 based on the date when suicide was committed. It provides information including the number of suicides by age, gender, employment status, site, and date of committing suicide. The data set includes a total of 214,855 suicides, accounting for 0.017% of the total population in 1848 Japanese cities (or districts in the metropolises of Tokyo, Osaka, and Kyoto) from 2013 to 2022, with a monthly average of 1790 suicides nationwide (Table S2 in Multimedia Appendix 1).

### Application of Deep Learning Models

We used an advanced deep learning model, BERT [21], using natural language processing to detect tweets with actual suicide ideation and thought. Compared with other deep learning models (eg, convolutional neural network, or long short-term memory), BERT presents state-of-the-art results in a wide variety of natural language processing tasks and can be pretrained to deal with Japanese texts [21].

### Generation of Training Data Sets via Human-in-the-Loop Modelling

We commenced with forging a training data set for the BERT model following a human-in-the-loop procedure. First, we implemented a filtering procedure to refine tweets with actual suicide risks via manual scanning by human efforts given that the original data set (containing a total of 62,083,647 tweets) retrieved using suicide-related keywords would inevitably contain tweets that might not obtain actual suicide ideation and thoughts. For example, a tweet of “suicide is not good” contains “suicide” but is not a suicide-risk tweet. This manual scanning procedure took 3 months from October 2022 to January 2023 by 2 research assistants who are native in the Japanese language. Through training workshops and cross annotation and calibration, these 2 research assistants largely achieved the same standard to judge suicide-risk tweets. Second, we randomly selected 30,000 tweets from the original keyword-filtered data set (ie, 0.05% of the total 62,083,647 tweets) to forge an initial training data set. The research assistants manually scanned these 30,000 tweets and annotated suicide-related tweets as positive samples if they indicated actual suicide risk or as negative samples if not. As a result, 7.52% (2257 out of 30,000 tweets) were identified as “positive”—tweets with actual suicide risks—whereas the rest were “negative,” non–suicide-risk tweets. This ratio of actual suicide-risk tweets over the total seems to be low but is reasonable and consistent with existing studies [22]. The labeled 2257 suicide-related tweets were used to train a BERT model. Third, the trained BERT model was used to identify the suicide-risk tweets, calibrated and validated again by the research assistants. As a result, 5734 tweets were detected as “positive,” and the model was trained again. A total of 30,861 tweets (7991 suicide-risk tweets and 21,272 non–suicide-risk tweets) were used to train the model, including 6393 suicide-risk tweets and 13,815 non–suicide-risk tweets in the training data set and 1598 suicide-risk tweets and 1598 non–suicide-risk tweets in the test data set. After 10 loops of training, we used the measures of precision, recall, and *F*_1_-score as the most widely used indicators to evaluate modelling performance and decide whether the BERT model had been well trained and could be applied to the entire data set. As a result, the values of precision, recall, and *F*_1_-score were 0.92, 0.80, and 0.85, respectively, indicating a sound and reliable modelling performance to classify suicide-risk tweets [12]. After the training of the BERT model, it was applied to the entire data set with the return of binary labels for each tweet, with 1 indicating suicide-risk tweets and 0 indicating non–suicide-risk tweets.

### Validating Twitter-Identified Suicide Risks With Actual Suicide Statistics

We compared between Twitter-identified suicide risks and actual suicide statistics in both the temporal and spatial dimensions via a Pearson correlation as the baseline, a smooth-window correlation, and a time lagged cross correlation. In the temporal dimension, we created a range of measures for Twitter-identified suicide risks to compare with the actual suicide records measured as the proportion of suicide deaths over the total population. Such measures included (1) the proportion of positive suicide-risk tweets over total tweets (including and excluding retweets separately); (2) the same proportion as (1) but with 2020 data excluded given that the outbreak of COVID-19 in 2020 may have brought a systematic interruption to the human society as a whole and influenced the behavior of suicide to some degree [23]; (3) the same proportion as (1) but with a certain time lag (ie, 1, 2, and 3 months) when correlating with actual suicide records, based on the observation from existing studies [14] that suicide behaviors and ideation might be influenced by celebrities on social media (ie, mimicking the suicide of celebrities after a certain period of time); and (4) the same proportion as (1) but manipulated via a smooth-window approach, that is, taking the average value of a certain month as well as its preceding and following months (ie, 3, 5, and 7 months) to reduce the impact of specific events (eg, natural disasters or public crises) on suicide, given that such events may have caused sharp changes in the suicidal trend. In the graphing process, we normalized these proportional measures by setting the first month, January 2013, as the baseline and dividing the measures in other months to the baseline for better comparison purposes.

In the spatial dimension, we commenced with mapping geotweets based on their latitude and longitude or the name of a city (ie, converted to the centroid of that city) to generate a point data layer containing the locations of all geotweets posted in the 10-year period. We then overlayed this point data layer with the polygon layer containing the boundaries of 1848 Japanese cities and 47 prefectures to generate the total number of geotweets within each city and prefecture. These data were then used to assess its correlation with the number of actual suicide records at the city and prefecture levels. We visualized the spatial pattern of geotweets and actual suicide records using the quintile classification in ArcGIS Pro 2.8 (Esri), with cities in the top quintile (ie, the top 80%-100% values of the data set) being identified as high suicide-risk areas. Herein, we compared the raw counts of geotweets and actual suicide records rather than the proportional measures as we did previously because (1) the total number of population and total tweets posted by city and by month is not available and (2) visualizing spatial patterns by the quintile classification is a relative approach by comparing values within one data set to identify hot spots (high-risk areas), and thus, it is less sensitive to the measure of proportions using the total population and total tweets.

### Ethical Considerations

This study does not involve human subjects at the individual level; thus, it is not subject to ethnical clearance. Tweets data retrieved via the Twitter API were deidentified, and we removed users’ ID and any identifiable information during the data-scanning process. All analyses were conducted at the aggregated population level based on sampling counts. All data saved in the project repository can be only accessed by research members.

## Results

### Temporal Comparison Between Twitter-Identified Suicide Risks and Actual Suicide Records

**Figure 1:**
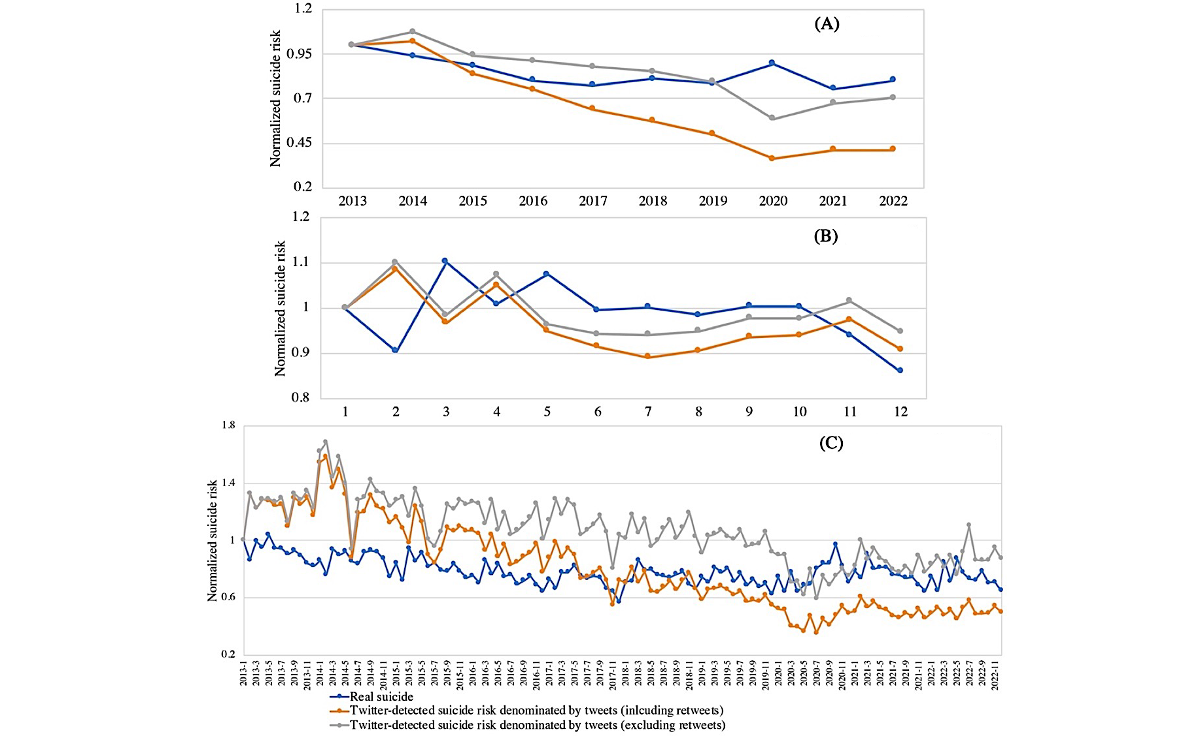
Comparison of actual suicide and suicide risks detected from Twitter (A) by year, (B) by month, and (C) by year and month.

The temporal comparison between Twitter-identified suicide risks and actual suicide records by year (Figure 1A) shows that the overall trend of Twitter-identified suicide risks and actual suicide were largely consistent—decreasing from 2014 to 2017 and slightly increasing from 2021 to 2022—although actual suicide records experienced an increase from 2019 to 2020 while Twitter-identified suicide risks were observed to decrease. A comparison by month (Figure 1B) shows a time-lag pattern in which the peak of Twitter-identified suicide risks appeared in February and April—1 month earlier than the peak of actual suicide records in March and May. If the actual suicide records are pushed 1 month forward, the overall trend of both data sources largely overlap, although they vary from October to November. We can also witness a time-lag discrepancy between Twitter-identified suicide risks and actual suicide by month and year (Figure 1C), although substantial fluctuations are observed over months. There is clear evidence of W-shape variations, for example, appearing from January to May in 2015, 2016, and 2018; from July to December in 2019; from January to May 2021; and from February to July 2022—where the line of actual suicide records (in blue) overlaps with that of Twitter-identified suicide risks (in orange or grey) if blue line is moved 1 month earlier.

**Figure 2:**
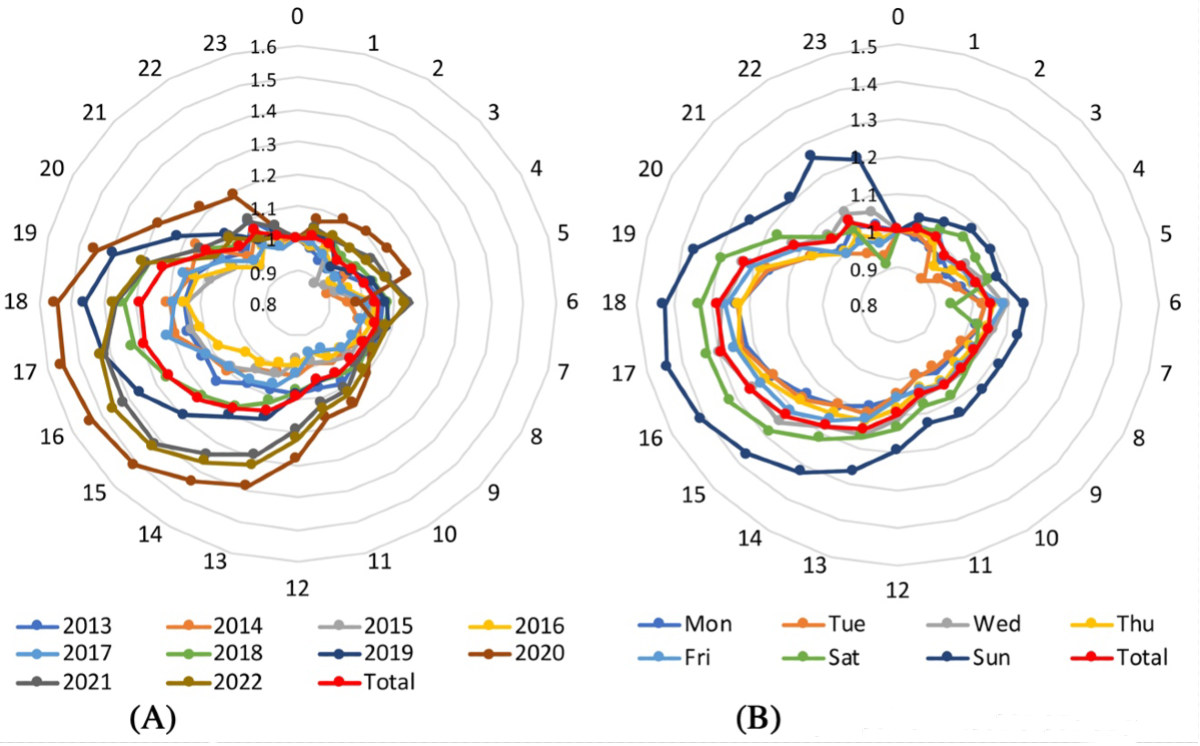
Twitter-identified suicide risks (A) by hour and year and (B) by hour and weekday.

We further examined the Twitter-identified suicide risks at a finer level (ie, by weekday and hour; Figure 2), which could not be revealed by the monthly ground-truth suicide records. The hour-by-year pattern (Figure 2A) and the hour-by-weekday pattern (Figure 2B) show that Twitter-identified suicide risks were at a relatively lower level after midnight (ie, from 1 AM to 5 AM) while displaying a higher level in the afternoon (ie, 4 PM to 6 PM). We speculate that such an hourly pattern reflects the suicide reality to some degree, although it may be partially explained by the variation of active Twitter users as fewer tweets are posted after midnight. In addition, the suicide risk in 2020, when the COVID-19 outbreak started, was obviously higher than that in other years (Figure 2A), regardless of the hour; the suicide risk in later years (eg, from 2019 to 2022) was higher than that in early years (eg, from 2013 to 2018). The suicide risk on Sundays and Saturdays (Figure 2B) was obviously higher than that on weekdays, possibly because the actual suicide statistics in Japan shows that home is the most frequent place where people commit suicide [20] and people have higher chances to stay at home over weekends.

**Figure 3:**
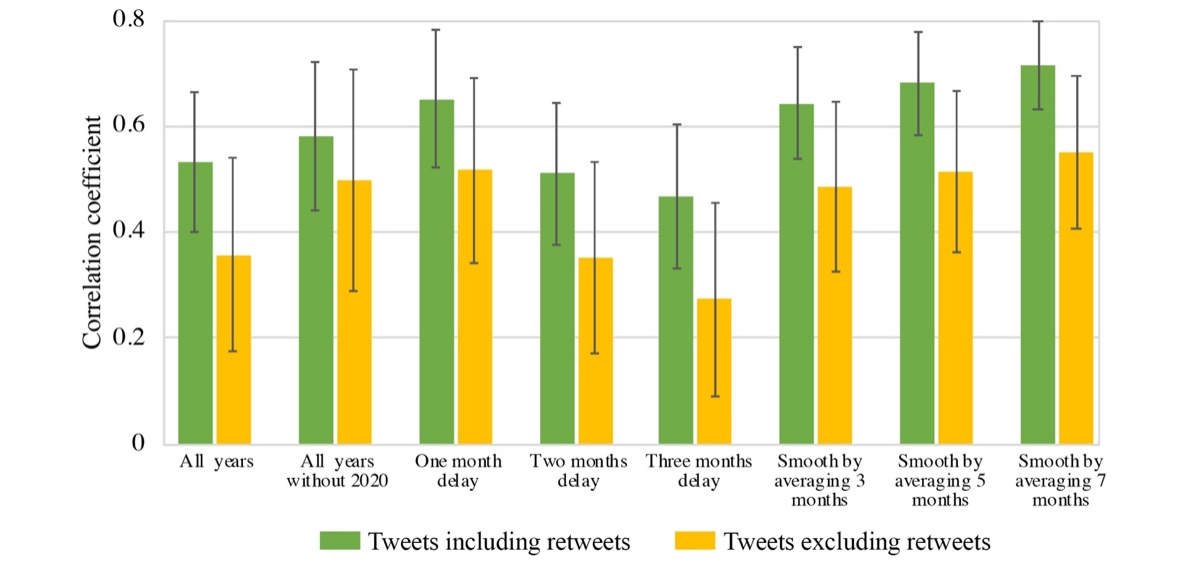
Correlation coefficients between Twitter-identified suicide risks and actual suicide records in different scenarios (detailed statistics are provided in Table S4 in Multimedia Appendix 1).

The time-lag effect observed previously was tested statistically through the correlation analysis (Figure 3 and Table S4 in Multimedia Appendix 1). “All years” indicates the correlation between Twitter-identified suicide risks and actual suicide records (0.533 and 0.361 for tweets including and excluding retweets, respectively; *P*<.001), serving as the baseline for comparison with other scenarios. By excluding the year 2020 with the COVID-19 outbreak as a systematic interruption to human society, the correlation coefficient increased to 0.620 and 0.502 (*P*<.001). By advancing actual suicide records 1 month earlier, the correlation coefficient further increased to 0.652 and 0.521 (*P*<.001); however, such an increase was not observed for advancing actual suicide records 2 or 3 months earlier. This finding reflects the potential existence of a 1-month time lag between the time when suicide-risk tweets were posted and the time when suicides were committed. By smoothing the data with averaged values across 3, 5, and 7 months, we observed much-improved correlation coefficients (maximum up to 0.716; *P*<.001) compared to “all years,” possibly due to the fact that data fluctuations across months are reduced by the smoothing method, revealing that the smoothed temporal trends of suicide risks identified by 2 data sets are highly correlated.

### Spatial Comparison Between Twitter-Identified Suicide Risks and Actual Suicide Records

**Figure 4:**
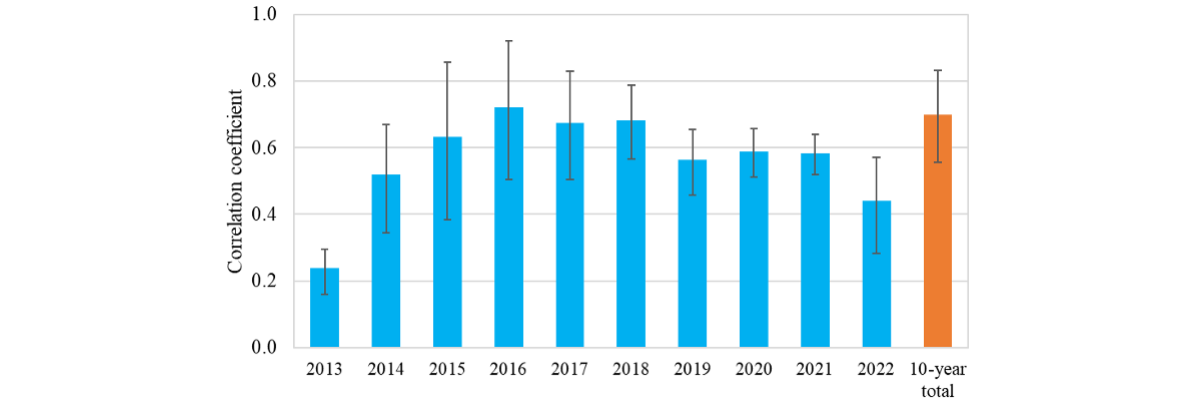
Correlation coefficients between suicide-risk geotweets and actual suicide records in every single year and in the 10-year period as a whole at the city level (detailed statistics are provided in Table S5 in Multimedia Appendix 1, and the correlation coefficients at the prefecture level are provided in Table S6 in Multimedia Appendix 1).

The correlation coefficients between geotweets and actual suicide records at the city level (Figure 4; detailed statistics are provided in Table S5 in Multimedia Appendix 1) were highest (0.721; *P*<.001) in 2016, followed by 2018 (correlation coefficient=0.683; *P*<.001) and 2017 (correlation coefficient=0.675; *P*<.001); meanwhile the correlation coefficient for the 10 years as a whole was 0.699 (*P*<.001), which is reasonably high, indicating that the cities where suicide-risk geotweets were concentrated are highly correlated with where actual suicides were committed. Moreover, the correlation coefficients between geotweets and actual suicide records at the prefecture level in each single year ranged from 0.725 in 2013 to 0.956 in 2016, and it was 0.945 for the 10 years as a whole (*P*<.001; Table S6 in Multimedia Appendix 1)—much higher than that at the city level. We also compared the correlation between geotweets and population density as well as between actual suicide and population density, with the majority of coefficients being below 0.4 (*P*<.001; Table S5 in Multimedia Appendix 1), indicating that population density has a minor interruption to the preceding correlation analysis.

**Figure 5:**
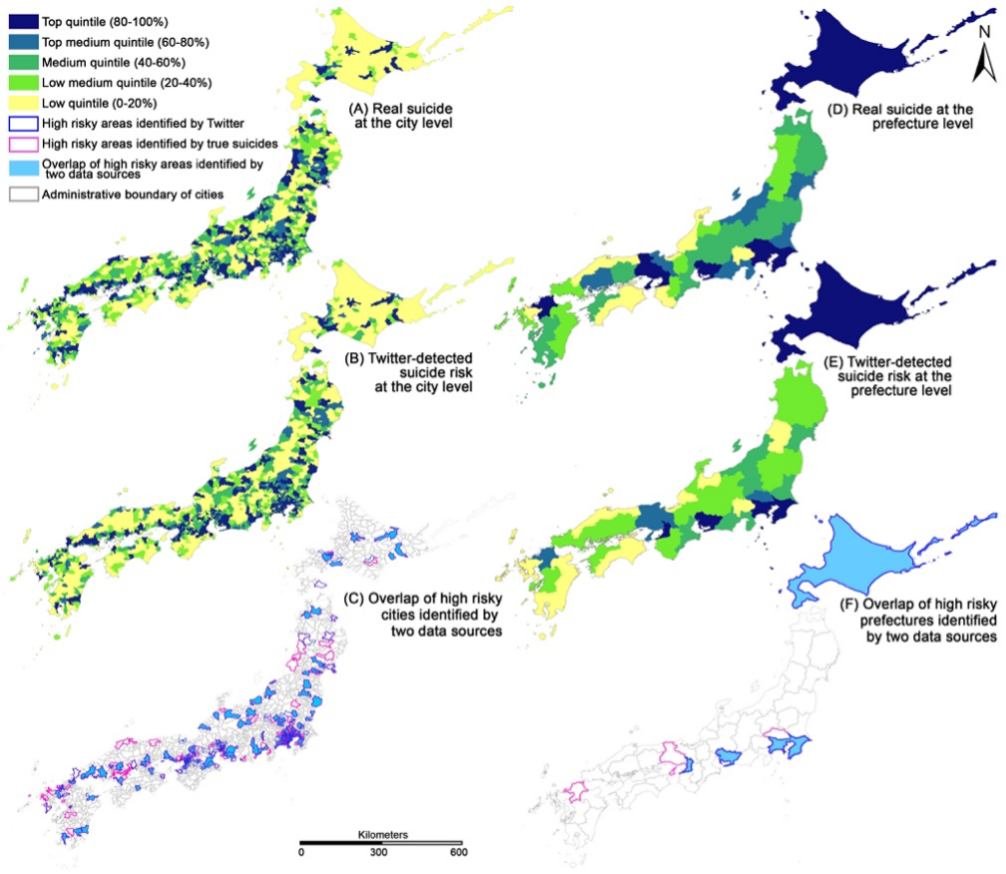
Spatial patterns of actual suicide records, Twitter-detected suicide risks via geotweets, and the overlap of high suicide risky areas identified by 2 data sources, at the city and prefecture level.

We further visualize the spatial pattern of areas with high concentrations of suicides (dark blue areas with the top quintile in Figure 5) based on the 10-year data as a whole, at the city and prefecture levels. The cities with high concentrations of suicide risks identified by geotweets and actual suicide records largely overlapped (Figure 5C)—196 out of 267 cities, accounting for 73.5% of high concentrations of suicides (or suicide risks) by both data sets. Although the overlapped prefectures identified as high risks of suicide by both data sources included the metropolises of Tokyo and Osaka and the prefectures of Chiba, Aichi, Kanagawa, and Hokkaido (Figure 5F), the proportion of the areas where suicides were highly concentrated over all cities in a prefecture (Table 1) was largest in Tokyo metropolis (29/62, 47%), followed by Kanagawa prefecture (25/56, 45%), Osaka metropolis (17/70, 24%), and Aichi prefecture (16/69, 23%).

**Table 1:** Proportion of cities with high concentrations of suicides over the total in a certain prefecture.

Prefecture	Value, n/N (%)
Tokyo (東京都)	29/62 (46)
Kanagawa (神奈川県)	25/56 (44)
Osaka (大阪府)	17/70 (24)
Aichi (愛知県)	16/69 (23)
Saitama (埼玉県)	12/72 (16)
Hyōgo (兵庫県)	8/49 (16)
Yamaguchi (山口県)	3/19 (15)
Chiba (千葉県)	8/59 (13)
Tochigi (栃木県)	3/25 (12)
Shizuoka (静岡県)	5/43 (11)
Fukuoka (福岡県)	8/72 (11)
Kyoto (京都府)	4/36 (11)
Niigata (新潟県)	4/37 (10)
Mie (三重県)	3/29 (10)
Miyagi (宮城県)	4/39 (10)
Hiroshima (広島県)	3/30 (10)
Ibaraki (茨城県)	4/44 (9)
Gunma (群馬県)	3/35 (8)
Miyazaki (宮崎県)	2/26 (7)
Okayama (岡山県)	2/30 (6)
Toyama (富山県)	1/15 (6)
Fukui (福井県)	1/17 (5)
Kagawa (香川県)	1/17 (5)
Ōita (大分県)	1/18 (5)
Ishikawa (石川県)	1/19 (5)
Fukushima (福島県)	3/59 (5)
Aomori (青森県)	2/40 (5)
Ehime (愛媛県)	1/20 (5)
Saga (佐賀県)	1/20 (5)
Okinawa (沖縄県)	2/41 (4)
Hokkaido (北海道)	9/188 (4)
Gifu (岐阜県)	2/42 (4)
Yamanashi (山梨県)	1/27 (3)
Kōchi (高知県)	1/34 (2)
Yamagata (山形県)	1/35 (2)
Nagano (長野県)	2/77 (2)
Nara (奈良県)	1/39 (2)
Kagoshima (鹿児島県)	1/43 (2)

## Discussion

### Principal Findings

Our study contributes to a 10-year investigation of suicide risks identified through social media in Japan, via a human-in-the-loop approach and an advanced deep learning model for natural language processing and tweet classification. The procedure of data collection (tweets over 10 years posted from January 2013 to December 2022), human scanning, data analysis, and modelling occurred from October 2022 to February 2023. We corroborated that the Twitter-identified suicide risks largely align with actual suicidal facts regarding their timing and location. More specifically, Twitter-identified suicide risks and actual suicide records were temporally correlated by month in the 10 years from 2013 to 2022 (correlation coefficient=0.533; *P*<.001); by advancing actual suicide records 1 month earlier, the correlation coefficient further increases to 0.652—reflecting the 1-month time lag between Twitter-identified suicide risks and actual suicide records. They were also spatially correlated by city with correlation coefficients of 0.699 and 0.945 (*P*<.001) for the 10 years as a whole at the city and prefecture levels, respectively. In all, 196 cities overlapped in the risk identification by tweets and actual suicide records, accounting for 73.5% of the cities in the top quintile of suicide risks classified at the national level.

### Comparison With Prior Work

Our study contributes to a growing body of research that harnesses social media expression in public health surveillance or infoveillance. More importantly, our study focuses on Japan, a super-aging society where the suicide rate increased significantly in the past 20 years [24], threatened all age groups—people aged 15-34 years with suicide as the top-leading cause of death [2] as well as the rapidly aging group older than 65 years who faces more disruptive challenges than the younger generation [25]. However, it is hard for care providers to monitor life conditions moment by moment for people with suicidal thoughts. They may not directly express their suicidal thoughts to friends or families because of an array of reasons, so social media may become the vent. Our findings on the correlation between Twitter-identified suicide risk and actual suicide behaviors indicate the potential existence of negative emotional contagion (people emotionally affect surrounding ones) in local or web-based communities, as observed in previous studies [26,27]. Our study supplements another layer that suggests that emotion contagion may transmit from web-based to offline space. Therefore, an early warning about the suicidal expression on social media and its correlation with cities and regions may help health care institutions to identify those high-risk areas, hence allocating the community sources in the right direction. Additionally, our research goes beyond prior social media–related studies that usually only adopt the platform as the data source [28,29] or do not involve actual suicide cases [14]. Integrating ground-truth suicide data with Twitter-identified data provided a more comprehensive landscape to explore the relationship between social media expression and its surveillance accuracy—the latter being essential for health intervention, considering that false alarms may destroy public trust.

### Policy Implications

The findings of our study have far-reaching public health implications for suicide prevention and intervention in and beyond Japan. First, we provide a novel approach via advanced natural language processing techniques to identifying and monitoring suicide risks at a population level in near real time, complementing traditional surveillance systems such as vital statistics and hospital records. It can be readily applied in different geographic contexts and languages, to address the voice of the “silent majority” in broader health initiatives and to predict suicide in countries that do not have reliable suicide statistics. Second, we show that statistically, social media data can serve as a valuable source of information for suicide prevention and intervention efforts [30,31]. The temporal and spatial patterns of suicide revealed in our study can inform the timing and location of suicide prevention strategies, such as targeting high-risk periods and areas for suicide prevention campaigns and outreach. For example, suicide prevention efforts in Japan may be particularly effective in addressing suicide risk in February and April when Twitter-identified suicide risks are at their peak. Additionally, consistent with prior research [32,33], identifying specific cities and prefectures with high concentrations of suicide risks can inform the allocation of resources for suicide prevention and intervention efforts at a regional level. Third, it is important to note that social media data do not replace traditional suicide surveillance systems but act as an alternative source to enrich the holistic picture of suicide. In practice, ethical considerations need to be considered, such as ensuring the privacy and consent of social media users [34].

### Limitations

Several limitations of this study deserve to be mentioned. First, we acknowledge the limited representativeness of social media data, as numerous studies have pointed out the rooted data biases toward certain demographics (or characteristics) and geographic locations. Second, the involved suicide-related keywords might not be comprehensive due to the variance of suicide-related expressions on social media. We acknowledge that tweets with suicidal risks expressed in different expressions might be excluded from our study. However, we are confident that the keywords used in this study were able to capture the majority of suicide-related tweets. Third, the definition of positive samples, which refers to tweets that indicate a risk of suicide, was manually defined and, therefore, is subject to a certain level of ambiguity. Thus, the repetitive model training process (ie, the human-in-the-loop strategy) might propagate such ambiguity toward the final classification. Fourth, we acknowledge that tweets with suicidal risks might not reflect the actual suicidal intention of users. In this study, we aimed to align suicide risks detected on social media with actual suicidal behaviors in a statistical manner, whereas linking suicidal expressions with suicidal intentions is beyond the scope of our study.

### Conclusion

In conclusion, our study provides new and valuable insights into the temporal and spatial patterns of suicide risks identified through social media data in Japan and corroborates the alignment between social media–identified suicide risks and actual suicide behaviors through the 10-year evidence. Our findings have important public health implications for suicide prevention and intervention efforts and highlight the potential of advanced natural language processing techniques for suicide surveillance on social media. By using newly emerging techniques and data sources in conjunction with traditional surveillance systems, we can work toward a comprehensive and effective approach to suicide prevention in Japan and beyond, especially in countries without reliable suicide statistics.

## Appendix

Multimedia Appendix 1 List of suicide-related keywords drawn from existing literature in English and Japanese, statistical summary of real suicide records, statistical summary of retrieved tweets data, correlation matrix between real suicide and Twitter-detected suicide risks, and correlation coefficients between geotweets and actual suicide records at the city and prefecture levels.

## Abbreviations

API: application programming interface
BERT: Bidirectional Encoder Representations from Transformers

## Data Availability

Due to the confidential concern, tweet data are not allowed to be shared with the public. Data at the aggregated level can be authorized via contact with the corresponding authors depending on research purposes. The actual suicide statistics are public available via the government website [20].

## References

[1] (2019). Suicide data. World Health Organization.

[2] (2020). Suicide rate for minors highest ever in Japan. Nippon.

[3] Nock MK, Borges G, Bromet EJ, Alonso J, Angermeyer M, Beautrais A, Bruffaerts R, Chiu WT, de Girolamo G, Gluzman S, de Graaf R, Gureje O, Haro JM, Huang Y, Karam E, Kessler RC, Lepine JP, Levinson D, Medina-Mora ME, Ono Y, Posada-Villa J, Williams D (2008). Cross-national prevalence and risk factors for suicidal ideation, plans and attempts. Br J Psychiatry.

[4] Richards JE, Whiteside U, Ludman EJ, Pabiniak C, Kirlin B, Hidalgo R, Simon G (2019). Understanding why patients may not report suicidal ideation at a health care visit prior to a suicide attempt: a qualitative study. Psychiatr Serv.

[5] Jashinsky J, Burton SH, Hanson CL, West J, Giraud-Carrier C, Barnes MD, Argyle T (2014). Tracking suicide risk factors through Twitter in the US. Crisis.

[6] Gunn JF, Lester D (2015). Twitter postings and suicide: an analysis of the postings of a fatal suicide in the 24 hours prior to death. Suicidologi.

[7] Morese R, Gruebner O, Sykora M, Elayan S, Fadda M, Albanese E (2022). Detecting suicide ideation in the era of social media: the population neuroscience perspective. Front Psychiatry.

[8] Robinson J, Cox G, Bailey E, Hetrick S, Rodrigues M, Fisher S, Herrman H (2016). Social media and suicide prevention: a systematic review. Early Interv Psychiatry.

[9] Pourmand A, Roberson J, Caggiula A, Monsalve N, Rahimi M, Torres-Llenza V (2019). Social media and suicide: a review of technology-based epidemiology and risk assessment. Telemed J E Health.

[10] Castillo-Sánchez Gema, Marques G, Dorronzoro E, Rivera-Romero O, Franco-Martín Manuel, de la Torre-Díez Isabel (2020). Suicide risk assessment using machine learning and social networks: a scoping review. J Med Syst.

[11] Macrynikola N, Auad E, Menjivar J, Miranda R (2021). Does social media use confer suicide risk? a systematic review of the evidence. Comput Hum Behav Rep.

[12] Coppersmith G, Leary R, Crutchley P, Fine A (2018). Natural language processing of social media as screening for suicide risk. Biomed Inform Insights.

[13] Sueki H, Ito J (2015). Suicide prevention through online gatekeeping using search advertising techniques: a feasibility study. Crisis.

[14] Ueda M, Mori K, Matsubayashi T, Sawada Y (2017). Tweeting celebrity suicides: Users' reaction to prominent suicide deaths on Twitter and subsequent increases in actual suicides. Soc Sci Med.

[15] Taira K, Hosokawa R, Itatani T, Fujita S (2021). Predicting the number of suicides in Japan using internet search queries: vector autoregression time series model. JMIR Public Health Surveill.

[16] (2023). Most used social media platforms in Japan as of 3rd quarter 2022. Statista.

[17] Homan C, Johar R, Liu T, Lytle M, Silenzio V, Alm C (2014). Toward macro-insights for suicide prevention: analyzing fine-grained distress at scale.

[18] Sueki H (2012). Relationship between suicide rate and use of search engines on the internet. Article in Japanese. Journal of Health and Welfare Statistics.

[19] (2022). BCCWJ Word List. Center for Language Resource and Development.

[20] (2022). Suicide statistics: local suicide basics. Article in Japanese. Ministry of Health Labour and Welfare.

[21] Tohoku NLP Group (2022). BERT base Japanese version 2. Hugging Face.

[22] Cheuk CT, Huan N, Cai R, Zhang J, Li Z, Li X (2023). Evaluation of artificial neural networks in natural language processing to identify suicide-risk messages on Twitter. JMIR Preprints. Preprint posted online on September 8, 2022.

[23] Tanaka T, Okamoto S (2021). Increase in suicide following an initial decline during the COVID-19 pandemic in Japan. Nat Hum Behav.

[24] Muramatsu N, Akiyama H (2011). Japan: super-aging society preparing for the future. Gerontologist.

[25] de Leo D (2022). Late-life suicide in an aging world. Nat Aging.

[26] Goldenberg A, Gross JJ (2020). Digital emotion contagion. Trends Cogn Sci.

[27] Tang J, Yu G, Yao X (2021). Emotional contagion in the online depression community. Healthcare (Basel).

[28] O'Dea B, Wan S, Batterham PJ, Calear AL, Paris C, Christensen H (2015). Detecting suicidality on Twitter. Internet Interv.

[29] Haque R, Islam N, Islam M, Ahsan MM (2022). A comparative analysis on suicidal ideation detection using NLP, machine, and deep Learning. Technologies.

[30] de Choudhury M, Kiciman E, Dredze M, Coppersmith G, Kumar M (2016). Discovering shifts to suicidal ideation from mental health content in social media.

[31] Spates K, Ye X, Johnson A (2020). "I just might kill myself": suicide expressions on Twitter. Death Stud.

[32] Han Y, Li H, Xiao Y, Li A, Zhu T (2021). Influential path of social risk factors toward suicidal behavior - evidence from Chinese Sina Weibo users. Int J Environ Res Public Health.

[33] Li H, Han Y, Xiao Y, Liu X, Li A, Zhu T (2021). Suicidal ideation risk and socio-cultural factors in China: a longitudinal study on social media from 2010 to 2018. Int J Environ Res Public Health.

[34] Conway M, O'Connor Daniel (2016). Social media, big data, and mental health: current advances and ethical implications. Curr Opin Psychol.

