# Supplementary material for: Public Surveillance of Social Media for Suicide Using Advanced Deep Learning Models in Japan: Time Series Study From 2012 to 2022
Source: J Med Internet Res. 2023 Jun 2;25:e47225. doi: 10.2196/47225 (PMC10276317; doi:10.2196/47225)
Supplement: Multimedia Appendix 1 [file jmir_v25i1e47225_app1.docx]

**Supplementary Materials**

**Table S1.** List of suicide-related keywords drawn from existing literature in English and Japanese

| **Suicide related keywords in English** | **Suicide related keywords in Japanese** |
| --- | --- |
| hang myself | 首吊り / 首を吊る / 首つり |
| intention to die | 死ぬ気 |
| hurt myself | 自分を傷つける |
| leave this world | この世を去る |
| deserve to die | 死ぬに値する |
| desire to end own life | 自分の人生を終わらせたいという願望 |
| kill myself | 自殺する |
| want death | 死にたい |
| self harm | 自傷 |
| take my life | 私の命を奪う |
| wanna/want to die | 死にたい / 死にたいです /自殺したい |
| my suicide note / letter | 私の遺書 |
| end my life | 私の人生を終わらせる |
| never wake up | 決して起きない |
| not worth living | 生きる価値がない |
| ready to jump | 飛び降りる |
| sleep forever | 永遠に眠る |
| Jump to a train | 電車に飛び込む |
| better off without me | 私がいないほうがいい |
| suicide plan | 自殺計画 |
| suicide pact | 自殺協定 |
| tired of living | 生きるのに疲れた |
| die alone | 一人で死ぬ |
| sleep forever | 永遠に眠る |
| my sad life | 私の悲しい人生 |
| stressed out | ストレスを感じる / ストレスで参っている |
| emotionally weak | 感情の起伏が激しい |
| hate myself | 私自身が嫌い |
| mentally weak | 精神的に弱い |
| Briquettes / burn myself | 練炭 /焼身 |
| Poison myself | 服毒 |
| suicide methods | 自殺方法 |
| suicide painless way to die | 自殺楽な死に方 /自殺楽 |
| group suicide join | 集団自殺募集 /集団自殺 / 自殺募集 |
| want to commit suicide | 自殺したい |
| want to end up my life | もう死にたい |
| painless suicide methods | 楽な自殺の方法 |
| painless suicide websites | 自殺サイト楽に死ねる方法 |
| suffering from living | 生きることがつらい |
| want to die help | 死にたい 助けて |
| painless suicide | 楽自殺 |
| commit suicide join | 自殺志願募集 |
| euthanasia methods | 安楽死方法 |
| most painless method to die | 一番楽に死ねる方法 |
| easy methods to die | 簡単に死ねる方法 |
| suicide manual | 自殺マニュアル /完全自殺マニュアル |
| erase myself | 消えたい |
| secure methods to die | 確実に死ねる方法 |
| why should I live / why do I live | 生きる意味が分からない |
| suicide methods easy | 自殺方法簡単 |
| depressed want to die | うつ 死にたい |
| suicide methods secure | 自殺方法確実 |
| suicide site | 自殺サイト |
| suicidal ideation | 自殺願望 |
| net suicide | ネット自殺 |

**Table S2.** Statistical summary of real suicide records

|  | **Number of**  **real suicides** | **Number of**  **the total population** | **% Suicides over**  **the total population** |
| --- | --- | --- | --- |
| 2013 | 25,550 | 127,445,000 | 0.0200 |
| 2014 | 23,978 | 127,276,000 | 0.0188 |
| 2015 | 22,626 | 127,141,000 | 0.0178 |
| 2016 | 20,448 | 127,076,000 | 0.0161 |
| 2017 | 19,764 | 126,972,000 | 0.0156 |
| 2018 | 20,652 | 126,811,000 | 0.0163 |
| 2019 | 19,959 | 126,633,000 | 0.0158 |
| 2020 | 22,673 | 126,216,430 | 0.0180 |
| 2021 | 19,030 | 125,681,593 | 0.0151 |
| 2022 | 20,175 | 125,500,000 | 0.0161 |
| Total | 214,855 | 1,266,752,023 | 0.0170 |

**Table S3.** Statistical summary of retrieved tweets data

|  | **Keyword-filtered tweets** | **Geotweets** | **All tweets with retweets** | **All tweets without retweets** |
| --- | --- | --- | --- | --- |
| 2013 | 5,526,468 | 1,755 | 3,315,944,026 | 2,854,835,957 |
| 2014 | 5,314,978 | 3,727 | 4,086,839,134 | 3,342,463,206 |
| 2015 | 6,052,841 | 4,822 | 4,590,710,863 | 3,525,939,056 |
| 2016 | 6,158,402 | 3,915 | 4,871,250,468 | 3,447,758,159 |
| 2017 | 6,171,874 | 2,599 | 5,385,308,552 | 3,377,993,762 |
| 2018 | 5,727,302 | 1,930 | 5,766,296,841 | 3,329,767,993 |
| 2019 | 6,108,751 | 1,682 | 6,476,868,044 | 3,485,726,192 |
| 2020 | 7,446,503 | 1,346 | 7,596,165,341 | 4,007,746,333 |
| 2021 | 6,488,511 | 925 | 7,886,376,633 | 4,164,907,786 |
| 2022 | 7,088,017 | 1,114 | 9,104,003,963 | 4,586,320,544 |
| Total | 62,083,647 | 23,815 | 59,079,763,865 | 36,123,458,988 |

**Table S4.** Correlation matrix between real suicide and Twitter-detected suicide risks

|  | **1** Twitter-identified suicide risks including retweets | **2** Twitter-identified suicide risks excluding retweets | **3** Actual suicide records |
| --- | --- | --- | --- |
| **2013-2022** |  |  |  |
| 1 | 1 |  |  |
| 2 | .937 | 1 |  |
| 3 | .533 | .361 | 1 |
| **Without 2020** |  |  |  |
| 1 | 1 |  |  |
| 2 | .933 | 1 |  |
| 3 | .620 | .502 | 1 |
| **1-month lag** |  |  |  |
| 1 | 1 |  |  |
| 2 | .938 | 1 |  |
| 3 | .652 | .521 | 1 |
| **2-month lag** |  |  |  |
| 1 | 1 |  |  |
| 2 | .937 | 1 |  |
| 3 | .512 | .356 | 1 |
| **3-month lag** |  |  |  |
| 1 | 1 |  |  |
| 2 | .937 | 1 |  |
| 3 | .468 | .277 | 1 |
| **Smooth of 3 months** |  |  |  |
| 1 | 1 |  |  |
| 2 | .950 | 1 |  |
| 3 | .644 | .488 | 1 |
| **Smooth of 5 months** |  |  |  |
| 1 | 1 |  |  |
| 2 | .955 | 1 |  |
| 3 | .682 | .518 | 1 |
| **Smooth of 7 months** |  |  |  |
| 1 | 1 |  |  |
| 2 | .959 | 1 |  |
| 3 | .716 | .553 | 1 |

**Table S5.** Correlation coefficients between geotweets and actual suicide records at the municipal level

|  | | **Actual suicide records** | | | | | | | | | | | **Pop density** |
| --- | --- | --- | --- | --- | --- | --- | --- | --- | --- | --- | --- | --- | --- |
|  |  | **2013** | **2014** | **2015** | **2016** | **2017** | **2018** | **2019** | **2020** | **2021** | **2022** | **Total** |  |
| **Geotweets** | **2013** | .239 | .233 | .243 | .240 | .234 | .231 | .230 | .230 | .236 | .217 | .241 | 0.147 |
|  | **2014** | .527 | .520 | .533 | .523 | .507 | .525 | .519 | .533 | .528 | .467 | .535 | 0.298 |
|  | **2015** | .625 | .629 | .633 | .623 | .613 | .627 | .623 | .636 | .627 | .548 | .639 | 0.331 |
|  | **2016** | .728 | .721 | .727 | .721 | .715 | .725 | .727 | .731 | .725 | .628 | .739 | 0.409 |
|  | **2017** | .679 | .686 | .691 | .686 | .675 | .679 | .678 | .690 | .678 | .576 | .696 | 0.389 |
|  | **2018** | .674 | .680 | .677 | .678 | .667 | .683 | .683 | .675 | .675 | .550 | .689 | 0.39 |
|  | **2019** | .561 | .561 | .561 | .567 | .547 | .566 | .564 | .558 | .563 | .457 | .571 | 0.344 |
|  | **2020** | .590 | .593 | .588 | .585 | .571 | .584 | .584 | .589 | .576 | .463 | .595 | 0.329 |
|  | **2021** | .589 | .595 | .595 | .586 | .568 | .583 | .596 | .596 | .584 | .494 | .600 | 0.321 |
|  | **2022** | .514 | .502 | .507 | .503 | .492 | .507 | .517 | .509 | .516 | .439 | .518 | 0.265 |
|  | **Total** | .687 | .686 | .692 | .686 | .672 | .685 | .685 | .691 | .686 | .588 | .699 | 0.391 |
| **Pop density** | | 0.382 | 0.39 | 0.39 | 0.38 | 0.376 | 0.397 | 0.395 | 0.414 | 0.412 | 0.272 | 0.401 | 1 |

Note: all coefficients are significant at p<0.01

**Table S6.** Correlation coefficients between geotweets and actual suicide records at the prefecture level

|  | | **Actual suicide records** | | | | | | | | | | | **Pop density** |
| --- | --- | --- | --- | --- | --- | --- | --- | --- | --- | --- | --- | --- | --- |
|  |  | **2013** | **2014** | **2015** | **2016** | **2017** | **2018** | **2019** | **2020** | **2021** | **2022** | **Total** |  |
| **Geotweets** | **2013** | .725 | .719 | .721 | .715 | .718 | .719 | .711 | .729 | .729 | .673 | .723 | 0.390 |
|  | **2014** | .916 | .912 | .917 | .913 | .909 | .915 | .918 | .927 | .931 | .803 | .918 | 0.533 |
|  | **2015** | .920 | .914 | .919 | .916 | .910 | .922 | .928 | .932 | .939 | .829 | .924 | 0.490 |
|  | **2016** | .953 | .953 | .959 | .956 | .950 | .955 | .958 | .960 | .969 | .809 | .957 | 0.554 |
|  | **2017** | .922 | .928 | .934 | .939 | .922 | .923 | .929 | .924 | .937 | .714 | .926 | 0.559 |
|  | **2018** | .926 | .937 | .940 | .943 | .930 | .927 | .932 | .925 | .936 | .700 | .929 | 0.569 |
|  | **2019** | .906 | .915 | .923 | .920 | .909 | .909 | .911 | .912 | .920 | .705 | .911 | 0.573 |
|  | **2020** | .888 | .895 | .901 | .906 | .887 | .890 | .896 | .885 | .898 | .645 | .890 | 0.542 |
|  | **2021** | .897 | .907 | .915 | .915 | .900 | .899 | .902 | .890 | .903 | .670 | .900 | 0.510 |
|  | **2022** | .921 | .924 | .934 | .929 | .919 | .930 | .933 | .927 | .937 | .756 | .927 | 0.536 |
|  | **Total** | .942 | .943 | .949 | .947 | .938 | .943 | .947 | .948 | .956 | .785 | .945 | 0.558 |
| **Pop density** | | 0.560 | 0.559 | 0.564 | 0.561 | 0.564 | 0.556 | 0.554 | 0.560 | 0.562 | 0.408 | 0.559 | 1 |
